# Reusable TiN Substrate for Surface Plasmon Resonance Heterodyne Phase Interrogation Sensor

**DOI:** 10.3390/nano10071325

**Published:** 2020-07-06

**Authors:** Ru-Jing Sun, Hung Ji Huang, Chien-Nan Hsiao, Yu-Wei Lin, Bo-Huei Liao, Yuan-Fong Chou Chau, Hai-Pang Chiang

**Affiliations:** 1 Department of Optoelectronics and Materials Technology, National Taiwan Ocean University, Keelung 202, Taiwan; s101011871@m101.nthu.edu.tw; 2 Taiwan Instrument Research Institute, National Applied Research Laboratories, Hsinchu 300, Taiwan; hjhuang@narlabs.org.tw (H.J.H.); cnhsiao@tiri.narl.org.tw (C.-N.H.); james722@tiri.narl.org.tw (Y.-W.L.); bohuei@tiri.narl.org.tw (B.-H.L.); 3 Centre for Advanced Material and Energy Sciences, Universiti Brunei Darussalam, Tungku Link, Gadong BE1410, Brunei; chou.fong@ubd.edu.bn; 4 Institute of Physics, Academia Sinica, Taipei 115, Taiwan

**Keywords:** refractive index, glucose solution, charge carrier density, surface plasmon resonance, heterodyne phase interrogation, sensitivities, long-term durability, TiN layer

## Abstract

A TiN-based substrate with high reusability presented high-sensitivity refractive index measurements in a home-built surface plasmon resonance (SPR) heterodyne phase interrogation system. TiN layers with and without additional inclined-deposited TiN (i-TiN) layers on glass substrates reached high bulk charge carrier densities of 1.28 × 10^22^ and 1.91 × 10^22^ cm^−3^, respectively. The additional 1.4 nm i-TiN layer of the nanorod array presented a detection limit of 6.1 × 10^−7^ RIU and was higher than that of the 46 nm TiN layer at 1.2 × 10^−6^ RIU when measuring the refractive index of a glucose solution. Furthermore, the long-term durability of the TiN-based substrate demonstrated by multiple processing experiments presented a high potential for various practical sensing applications.

## 1. Introduction

The detection and treatment or removal of harmful materials in water are essential for environmental protection. Typically, dissolved material in water can be detected using several methods such as absorbance spectroscopy, biological oxygen demand (BOD) [1,2] and chemical oxygen demand (COD) [3,4] evaluation, colorimetry [5], gas chromatography–mass spectrometry (GC–MS) [6], surface-enhanced Raman spectroscopy [7,8,9,10], electrochemical cyclic voltammetry (CV) [11,12,13], and electrical conductivity detection. Few biological agents, i.e., bacteria and viruses, can be detected through polymerase chain reaction (PCR) [14,15,16]. However, these methods may require reagents and instrumentation that are cost-intensive. They also are difficult in processing real-time and long-term applications for drinking water monitoring.

Fortunately, the dissolution of many biological or chemical materials typically leads to sudden alterations in the refractive index of water. Thus, real-time monitoring of the water refractive index potentially ensures the overall safety of drinking or effluent water. High-sensitivity sensors and methods of long-term durability are demanded for ultralow refractive index changes and high stability in water monitoring [17,18,19].

The total internal reflection (TIR) of light inside the surface of a prism is sensitive to the change of the surrounding material (e.g., air or liquid). The sensitivity of the refractive index detection can be improved further through a Kretschmann configuration [20,21,22,23,24,25,26]. A subsystem of a fixed small tank or chamber with inlet and outlet on the surface of a prism can be used to detect the refractive index of the flowing-through liquid. The detection prism surface is covered with an additional metal, thin film, which induces surface plasmon resonance (SPR) through TIR coupling of the incident light. Generation of SPR in the collective oscillation of free electrons at the metal–dielectric interface leads to a dip in the reflection spectrum. The so-called attenuated total reflection (ATR) method is based on ultrahigh sensitivity to the changes in the refractive index of the covering dielectric material, i.e., target solutions [21,27,28,29,30,31,32,33,34,35,36].

Moreover, nano roughness or sculptured structures on the thin film [37,38,39,40,41,42,43] further provides high-sensitivity, localized SPR modes and high specific surface area, which can lead to increased sensitivity to the refractive index of the covering material in many applications [25,27,44,45,46,47,48,49,50,51,52,53,54,55,56,57,58,59]. Nanoparticles, nanostructures, and thin films constructed with a metal material can also induce localized SPR under light illumination [25,27,44,45,46,47,48,49,50,51,52,53,54,55,56,57,58,59,60,61,62]. It is beneficial to fabricate metal nanoparticles or nanostructures in suitable sizes to detect SPR refractive indexes with high sensitivity at a specific wavelength of light. Peng et al. measured refractive index changes at a sensitivity of 6.3 × 10^−8^ RIU by using a Ag nanoparticle-based sensor [21].

Optical responses of array-structured nanomaterials or (random) photonic crystals can also be largely altered by changing surrounding materials [20,46,47,48,49,50,51,52,53,54,55,56,57,58]. The light transmission–absorption spectrum of metal 2D array nanostructures can effectively provide highly accurate refractive index measurements of the liquid sample [47,50,51,52,53,54,55]. Arrays are built using Ag and dielectric nanospheres or nanorods [50], Ag shell dielectric–core nanorods [47], metal–dielectric nanorods with or without a rotational angle [51], protruding metal nanorods (MNRDs) in a core–shell nanorod [52], MNRDs with connected veins [53], Ag-coated Si nanorods [54], and nanorods with crosshairs [55]. A metal–insulator–metal (MIM) sensor structure coupled with several Ag nanorod defects in a T-shape cavity [59] presents a relatively high sensitivity for refractive index sensing. Thus, a layer of vertical standing rods in the Kretschmann configuration can be useful as a sensing medium.

However, Ag has high chemical activity; oxidization of Ag nanoparticles can significantly reduce the sensitivity of refractive index measurements. Moreover, the deposited noble metal layers tend to peel off easily from the substrate due to low adhesion, and this is a disadvantage for long-term use. High-sensitivity plasmonic refractive index detection in the long-term monitoring of liquid requires metal-like materials with high chemical stability. The nanostructures of metal-like materials can further increase the sensitivity of the detection system. In 2010, Naik et al. applied hard TiN with high chemical stability and achieved a charge carrier density of approximately 10^22^ cm^−3^ [63,64]. In 2011, Chen et al. fabricated TiN with a charge carrier density of 6.6 × 10^22^ cm^−3^. The TiN layer has a high carrier concentration and low chemical activity, like metal [65]. In 2011, Kumar et al. presented an inclined deposition method to grow inclined crystal rods [66]. In general, TiN layers have high chemical and electrical stability that can be used for fabricating supercapacitors [67], lithium hosts in batteries [68], and electrochemical hydrogen storage [69]. Deposited TiN layers [63,64,65,66,67,68,69,70,71,72] of nanostructures are good plasmonic materials and can be used to fabricate novel sensors with long-term stability.

In practical setup, the plasmonic ATR sensing process is commonly categorized into four basic types: (i) angular interrogation (i.e., change of the resonant angle), (ii) intensity interrogation (i.e., change of reflectance at a fixed incident angle), (iii) wavelength interrogation (i.e., change of resonant wavelength at a fixed incident angle), and (iv) phase interrogation (i.e., the phase difference between P- and S-polarized light in the reflection spectrum) [22,27]. Among these, the phase interrogation technique provides the most sensitive measurements [28,29,30,31,32,33,34,35,36]. In our previous work, the wavelength of incident light was found to affect detection sensitivity in SPR temperature monitoring [30]. Moreover, SPR phase interrogation at the particular incident wavelength provided a high-resolution angular measurement and other applications [31,73,74,75,76,77].

In the Kretschmann configuration, the generated SPR mode is affected by the wavevector of incident light and material properties of the substrate, covering layer, and the SPR generating metallic layer. On fixing the material properties of the metallic layer and substrate, the material property, i.e., refractive index, of the covering layer can be resolved by the SPR or plasmonic optical response modes concerning various wavevectors of incident light. Angular and wavelength interrogation both introduce variations in the wavevector of incident light to resolve the refractive index of the covering material. Chen et al. [65] demonstrated that TiN has a relatively low charge carrier density compared with Ag and had a broader valley in the angular and wavelength interrogation measurements. This means that the lowest point of the valley cannot be identified clearly under external optical or electrical interference if the TiN layer is chosen to be the SPR generator in Kretschmann configuration measurements. The modified method suggested by Nelson et al. [28] (see Equation (4)), using slopes of the data curve to estimate the covering material’s refractive index, has presented good sensitivity [20,21,44] with Ag or other noble metals used in the SPR generator. The modified method only needs the metallic material to have enough charge carrier concentration to generate SPR and introduces variations in plasmonic reflections and phase differences for P- and S-polarized incident light. Based on this modification, the detection limit (RIU) of the modified phase interrogation technique is inversely proportional to the typically used definition of sensitivity (nm/RIU) in evaluating a plasmonic sensor. The detection limit refers to the lowest difference in measurements for the point-by-point conversion of the refractive index. It was used in this study to make sure it did not cause confusion with typically used “sensitivity.”

In this study, a TiN layer was deposited in compact standing crystal rods, which provided high-sensitivity refractive index measurements. An additional stacked layer of inclined-deposited TiN (i-TiN) nanorods can increase the specific surface area to contact the target solution, which results in a further enhanced sensitivity in experimental measurements. High chemical stability of the TiN layer facilitated consistent measurements in the cycling of clean-and-reuse experiments, indicating the long-term durability. Analytical solutions with multiple-layer reflectivity and phase differences based on the Fresnel equation were also acquired by illuminating light on a 46 nm TiN layer to cover liquids with various refractive indexes.

## 2. Experimental Setup

### 2.1. Sample Preparation

TiN and i-TiN thin layers were deposited on a Si substrate by using a radiofrequency (RF) magnetron sputter. The processing temperature of the substrate was 400 °C, and the starting pressure was 5 × 10^−6^ Torr. The RF source was maintained at 250 W under a flow of Ar (7 sccm) and N_2_ (5 sccm) air (237 K, 760 Torr).

**Figure 1:**
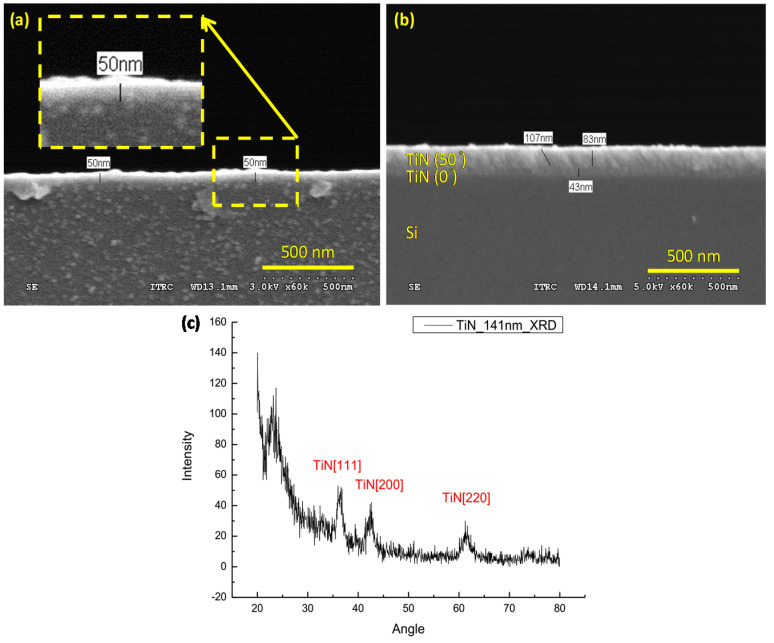
Scanning electron microscopy images of the TiN layer deposited on a Si substrate at various deposition angles: (**a**) 0° (50 nm) and (**b**) 0° (43 nm)/50° (83 nm). (**c**) X-ray diffraction measurement of deposited TiN (141 nm) on the Si substrate.

Sample Series 1 comprised normally grown TiN layers of various thicknesses, deposited on a BK7 glass slide (25 × 25 × 1 mm^3^), whereas Sample Series 2 comprised TiN(43 nm)/i-TiN layers, deposited on an identical glass slide. The thicknesses of i-TiN layers were changed using various deposition times and by tilting the substrate 50° during the sputtering process. The TiN (Figure 1a) and TiN/i-TiN (Figure 1b) thin layers deposited on the Si substrate were visualized through scanning electron microscopy.

The i-TiN layer has a higher specific surface area to contact the target solution and enhance the sensitivity of refractive index measurements. The smallest thickness of the homogeneous i-TiN layer fabricated by the inclined deposition method was 1.4 mm. X-ray diffraction analysis (Figure 1c) revealed that the deposited TiN layer (141 nm thick) demonstrated various signal peaks of typical crystal surfaces.

Table 1 presents the charge carrier concentrations of samples measured by a Hall effect measurement system (Accent HL5500PC) with various thicknesses of deposited TiN thin layers. Typically, the bulk carrier density (BCD) is calculated from the sheet carrier density (SCD) data following the definition BCD=SCD/Δh, where Δh is the thickness of the sample thin film. The deposited TiN layer on the glass substrate could achieve the highest bulk and sheet carrier densities up to 1.91 × 10^22^ cm^−3^ and 8.77 × 10^16^ cm^−2^, respectively. Thus, the deposited TiN layer could resemble Si, whereby it could generate SPR under light illumination. The additional i-TiN layer slightly reduced the bulk and sheet carrier densities but enhanced the precision of data acquisition in subsequent SPR heterodyne phase interrogation measurements (Table 1).

### 2.2. Experimental Setup

**Figure 2:**
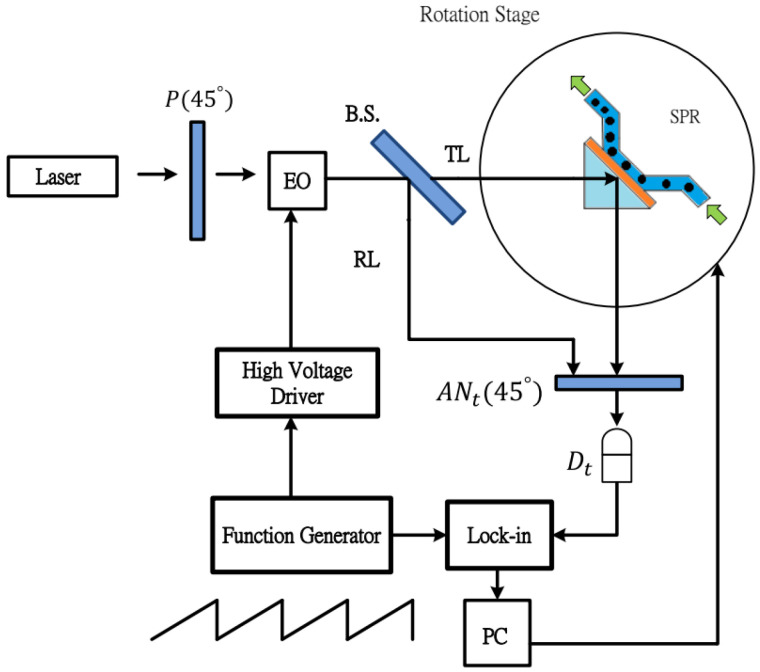
Schematic of the experimental setup in the Kretschmann configuration for refractive index measurements. P: polarizer, BS: beam splitter, AN: analyzer, D: detector, TL: test light, RL: reference light.

An SPR heterodyne phase interrogation system was constructed to detect the refractive index of a glucose solution flowing through a small liquid test cell (Figure 2). In the experimental setup, a polarizer defined the polarization of 1150 nm coherent light from a laser. The continuous polarized light was modulated into a jagged variation on illuminated intensity by an electro-optic (EO) modulator (ADP, NH_4_H_2_PO_4_, ConOptics) that was triggered by a function generator (DS345, Stanford Research System) and high-voltage driver (Model: 302, ConOptics). The beam splitter (BS) further divided the light into two light beams, namely test and reference light beams, with perpendicular propagation paths. The test beam illuminated the liquid test cell through a prism with the Kretschmann configuration [22,70]. Surface plasmons are only generated by illuminating P-polarized light in the deposited metal layer and should be affected by the refractive index of the covering test solution. The test glucose solutions of various concentrations can flow in the circular test flow cell (diameter, 20 mm; depth, 0.5 mm; volume, 0.157 mL), defined by an aluminum chamber and O-ring.

Both the reference beam and the output light from the testing unit also propagated through the analyzer, converted to an electrical test signal, and was sent to a lock-in amplifier. The reflectivity and locked-in phase difference were obtained by referencing the light-modulating electrical signal from the function generator and then translating it to the refractive index of the test solution.

In the experimental setup, the light intensity of the test beam measured using the detector can be expressed as

(1) $$I_{t}={\left|E_{t}\right|}^{2}=\frac{1}{2}\left(\frac{{\left|r_{p}\right|}^{2}+{\left|r_{s}\right|}^{2}}{2}+\left|r_{p}\right|\left|r_{s}\right|cos\left(\omega t+\phi_{p}-\phi_{s}\right)\right)$$

where $I_{r}$ and $I_{t}$ are the reference and the test beam intensities, respectively, both of which have the oscillated term cos(ωt). ω is the light intensity modulation frequency defined by a function generator and EO modulator. The refraction coefficients of polarized P-wave ($\left|r_{p}\right|$) and S-wave ($\left|r_{s}\right|$) are the ratio of the reflected wave’s electric field complex amplitude to that of the incident wave. Therefore, the light intensity-related term $\left|r_{p}\right|\left|r_{s}\right|$ and phase difference term (Δϕ) of P- and S-polarized light ($\Delta \phi =\phi_{p}-\phi_{s}$) can be extracted through calculation with $I_{r}$ and $I_{t}$ by using the lock-in amplifier. Furthermore, only the electromagnetic wave with an electric field that lies on the incident plane can induce SPR in the Kretschmann configuration and $\left|r_{s}\right|\sim 1$. The square of the readout value from the lock-in amplifier is ${\left(\left|r_{p}\right|\left|r_{s}\right|\right)}^{2}$, which is equal to the ratio of light reflected by the metal layer in experiments [44].

## 3. Results and Discussion

**Figure 3:**
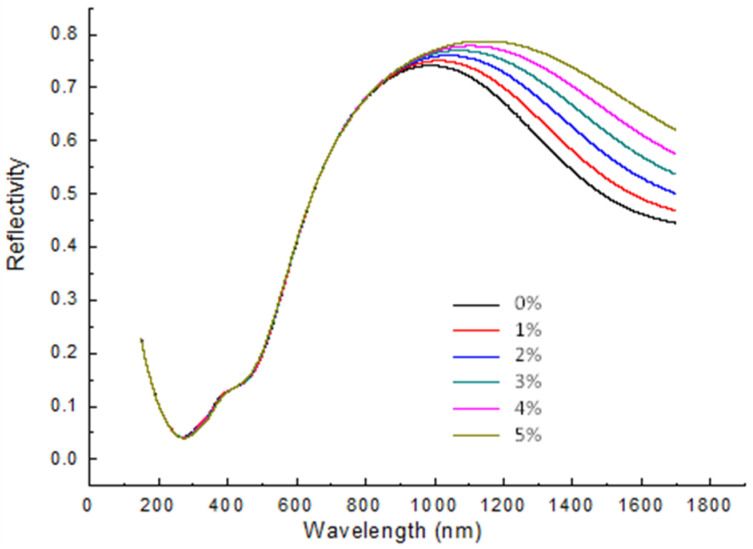
Numerical reflectivity calculations at various wavelengths of 49.7° oblique incident light for various glucose solution concentrations. The thickness of the deposited TiN layer is 46 nm.

**Figure 4:**
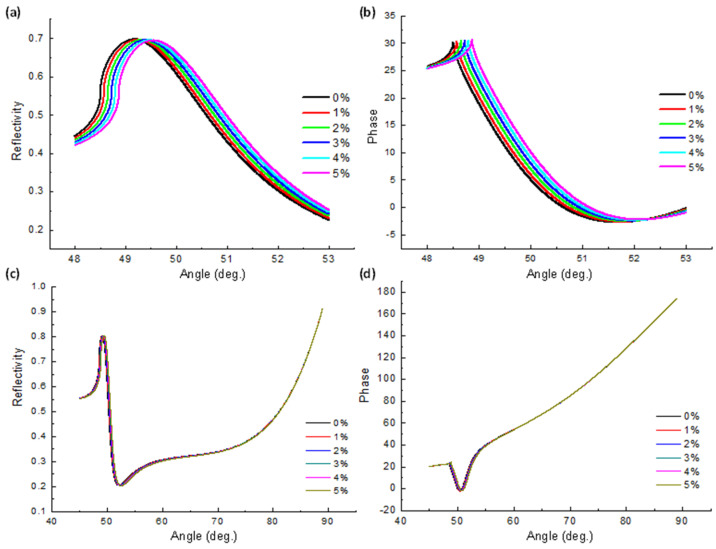
Numerical calculations of (**a**,**c**) reflectivity and (**b**,**d**) phase differences with various incident angles of 1150 nm incident light for various concentrations of glucose solution. The thickness of the deposited TiN layer is 46 nm.

Analytical solutions of multilayer reflectivity were used to acquire with various wavelengths (Figure 3), reflectivities (Figure 4a,c), and phase differences (Figure 4b,d) for various light incident angles based on the Fresnel equation for multiple optical reflections and transmissions; light was illuminated from the glass substrate to a 46 nm TiN layer covered with glucose solutions of various refractive indexes [44]. Theoretical estimation of reflection coefficient ($r_{ij}$) was acquired as inclined incident light illuminating from the i-th to j-th multilayers:

(2) $$r_{ij}=\frac{\left(Z_{i}^{q}+Z_{j}^{q}\right)}{\left(Z_{i}^{q}+Z_{j}^{q}\right)}$$

where q denotes the P- or S-wave. $Z_{i}^{P}=\varepsilon_{i}/k_{zi}$ and $Z_{i}^{S}=k_{zi}$. $\varepsilon_{i}$ is the dielectric constant of incident light for the i-th layer. $k_{zi}=\sqrt{k_{x}^{2}-\varepsilon_{i}k_{0}^{2}}=k_{0}\sqrt{\varepsilon_{i}-\varepsilon_{1}sin^{2}\theta }$. $k_{0}$ and $k_{x}$ are the wavevector and its component in the x-direction, respectively. θ is the illuminating angle of incident light. The multiple transmissions and reflections of the three layers (glass/TiN/glucose solution) can be simplified to acquire the reflection coefficient:

(3) $$r_{123}=\frac{\left(r_{12}+r_{23}e^{i2k_{z2}d_{2}}\right)}{\left(1+r_{12}r_{23}e^{i2k_{z2}d_{2}}\right)}$$

The subscripts 1, 2, and 3 denote layers of glass, TiN, and glucose solution, respectively. $d_{2}$ is the thickness of the TiN layer. The reflection coefficients are both complex numbers for P- and S- polarized light and can be expressed as $r_{p}=\left|r_{p}\right|e^{i\phi_{p}}$ and $r_{s}=\left|r_{s}\right|e^{i\phi_{s}}$, respectively.

The reflectivity with various wavelengths of oblique incident light at 49.7° for various concentrations of glucose solution [44] can be analytically resolved by the reflection coefficient above. The differences in reflectivity for various concentrations of glucose solution were not large for incident light wavelengths shorter than 850 nm. TiN is thus typically used to generate SPR with light longer than 850 nm [65,66]. With low chemical activity and strong mechanical hardness, deposited TiN layers [63,64,65,66,67,68,69] of nanostructures are good plasmonic materials with long-term stability.

**Figure 5:**
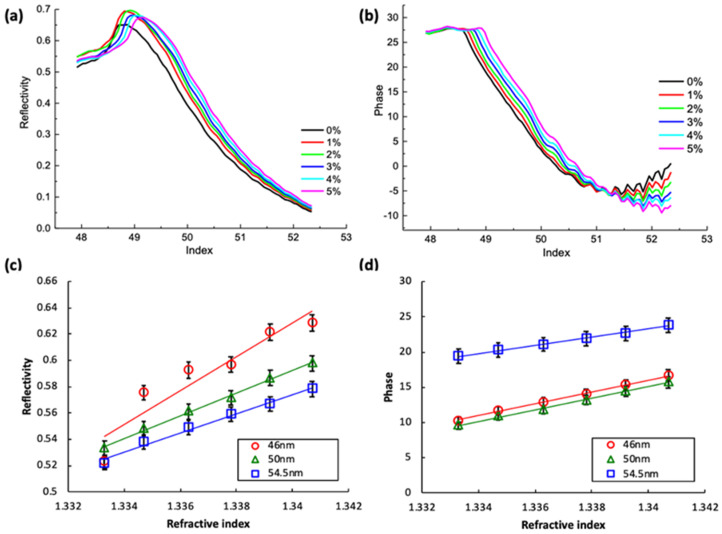
(**a**) Reflectivity and (**b**) phase differences with various incident angles for various concentrations of glucose solution. The thickness of the deposited TiN layer is 46 nm. (**c**) Reflectivity and (**d**) phase for various refractive indexes (real part) of glucose solution using samples with various TiN layer thickness.

**Figure 6:**
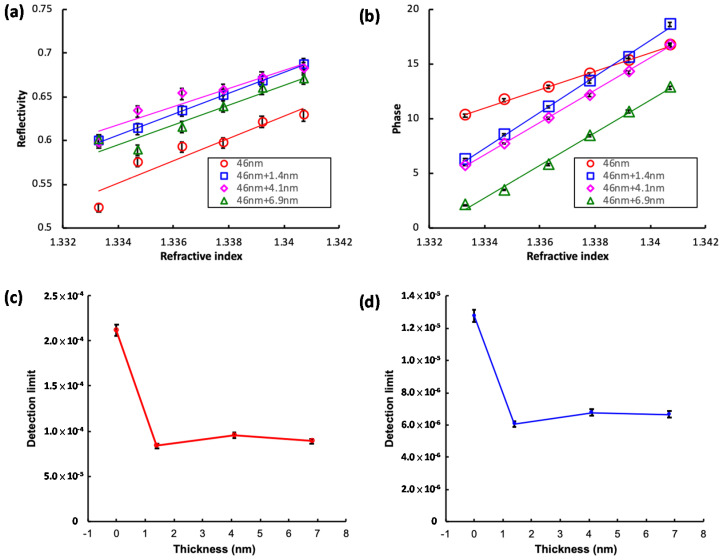
Dependence of (**a**) reflectivity and (**b**) phase on the various refractive indexes (real part) of glucose solutions on the various inclined deposition lengths of 46 nm of TiN layers deposited on glass. Dependence of detection limit of (**c**) reflectivity and (**d**) phase on the various thicknesses of additional TiN layers.

The reflectivity (Figure 4a,c) and phase difference (Figure 4b,d) in calculations delivered similar results to those in experimental measurements (Figure 5 and Figure 6). The wavelength of light in the analytical calculations was 1150 nm. The theoretical calculations showed that the reflectivity (Figure 4c) and phase difference (Figure 4d) presented relatively large variations for incident light illuminating at 45°–65°. Due to the limitation of the experimental setup processed with a prism, the experimental results were only acquired with incident angles at 48°–52° without loss of generality. The refractive index estimation was based on the slope of the data curve with incident light at around 49.7°.

In the theoretical calculation (Figure 4) and experimental measurements (Figure 5), the relatively higher value of reflectivity and phase difference close to 49° indicated the critical angle of TIR in Kretschmann configuration, which resembled results presented in Figure 5 in the reference work [65]. As the incident light angle further increased, the TIR coupling and SPR generation quickly decreased and experienced a sharp dip in reflectivity, which is typically seen in many plasmonic ATR sensing applications. The relatively high reflectivity at the critical angle is usually not as apparent when using a thin, metal film such as with a SPR generator. However, the TiN thin film has low charge carrier concentrations and has a relatively apparent, high reflectivity at the critical angle and broader dip or valley, indicating SPR response [65].

In experimental measurements, the various concentrations of glucose solution presented slightly shifted patterns of reflectivity (Figure 5a) and phase differences (Figure 5b) concerning different light illumination angles. The reflectivity (Figure 5c) and phase difference (Figure 5d) for 49.7° incident light were acquired and presented a linear agreement with the known refractive indexes of the glucose solution.

As shown in Table 1, the 46 nm deposited TiN layer exhibited the highest bulk and sheet carrier densities. Experimental results in Figure 5c suggest that the sample with the 46 nm TiN layer presented relatively high reflectivity. The results of phase difference measurements (Figure 5d) also showed that samples with 46 nm TiN layers demonstrated relatively high precision and stability. Therefore, the sample with the 46 nm TiN layer was chosen as the base for an additional i-TiN layer.

The additional i-TiN layers of various thicknesses further enhanced the reflectivity (Figure 6a,c) and precision of the phase difference (Figure 6b,d) measurements of the refractive indexes of variously concentrated glucose solutions. The additional 1.4 nm i-TiN layer improved the performance of the base 46 nm normally deposited TiN layer. In order to acquire the detection limit or smallest refractive index unit (RIU) ($\sigma_{n}$) resolvable in reflectivity and phase measurements, we followed the definitions of Nelson et al. [28] and our previous work [20,21,44] to consider the following quantity:

(4) $$\sigma_{n}=\left(\frac{\Delta n}{\Delta \phi }\right)\sigma_{\phi }$$

where Δn/Δϕ is the local slope of the refractive index n versus phase ϕ curve, and $\sigma_{\phi }$ is the finest resolution available, which is 0.01°, from the lock-in amplifier used in our experiments. The slopes Δn/Δϕ of the phase measurements for a 46 nm TiN layer with and without an additional 1.4 nm i-TiN layer were 6.1 × 10^−5^ and 1.2 × 10^−4^, respectively. Therefore, the acquired refractive index detection limit for glucose solution in the phase mode increased to 6.1 × 10^−7^ RIU with the addition of a 1.4 nm i-TiN layer. This was better than the 1.2 × 10^−6^ RIU obtained with the 46 nm TiN layer (Figure 6b).

The experimental results showed that the phase difference data (Figure 5d and Figure 6b) had higher consistency than that in the reflectivity (Figure 5c and Figure 6a) concerning various refractive indexes. The modification with Equation (4) presented higher stability of acquired data in phase difference measurements. Addition of the i-TiN layer further decreased the detection limit. The Nelson’s modification of phase difference measurements and additional i-TiN layer in combination presented the lowest detection limit of 6.1 × 10^−7^ RIU in our measurements.

Experimental results presented that use of the SPR heterodyne phase interrogation system with low chemical activity and a reusable TiN layer was beneficial for refractive index detection in the liquid phase. The protruded nanorods can have a larger specific surface area to contact the target solution and further enhance the sensitivity of refractive index measurements in the SPR heterodyne phase interrogation system [20]. However, addition of an inclined-deposited TiN layer in the nanorod array reduced the carrier concentration. The increase of the i-TiN layer thickness gradually reduced the detection limit in measurements.

**Figure 7:**
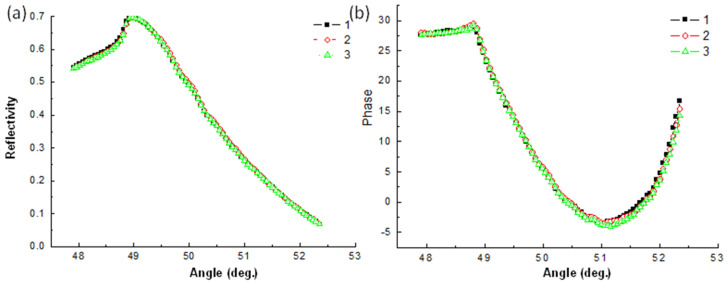
Reusability testing in three runs for (**a**) reflectivity and (**b**) phase measurements.

The TiN-based sensor also presented potential reusability in three repeated trials. Results for 5% glucose solution were highly similar among the three repeated experiments. The intervals after every run involved cleaning with acetone, methanol, and deionized water sequentially. The experimental data in reflectivity (Figure 7a) and phase (Figure 7b) measurements presented similar results in three experiment runs. The deposited TiN layer provided stability and was reusable in multiple processes of refractive index sensors. The typically used thin metal films (e.g., Ag or Au) for plasmonic applications are easily oxidized or peeled from the substrate, which makes them unsuitable for reuse or long-term storage. Thus, the high stability and reusability of the deposited TiN layer could increase the long-lasting durability of plasmonic refractive index sensors or measurements in future applications.

## 4. Conclusions

In this study, TiN with additional i-TiN thin layers was successfully deposited on a glass substrate by using the inclined deposition method. The bulk charge carrier densities of the deposited TiN layer with and without an additional i-TiN layer reached 1.28 × 10^22^ and 1.91 × 10^22^ cm^−3^, respectively. A home-built SPR heterodyne phase interrogation system was used for measuring the refractive index of glucose solutions. The experimental results presented good agreement with the Fresnel equation’s analytical solutions of multiple-layer reflectivity and phase differences. The refractive index of a liquid was resolved by using the SPR heterodyne phase interrogation method in the theoretical model. The detection limits of the sample solution using the 46 nm TiN layer with and without an additional 1.4 nm i-TiN layer were 6.1 × 10^−7^ and 1.2 × 10^−6^ RIU, respectively. The detection limit of the plasmonic ATR refractive index measurement using a TiN layer was lower than that using an Ag layer [44]. However, the reusability and low cost of the TiN layer provide high potential for use as practical plasmonic sensors for biosensing with potential long-term durability in future applications.

## Figures and Tables

**Table 1:** Charge carrier concentrations of samples with various deposited thicknesses of the TiN thin layer. The error of the measurement is smaller than 1%.

	Bulk Carrier Density (1/cm^3^)	Sheet Carrier Density (1/cm^2^)
Glass/TiN (46 nm)	1.91 × 10^22^	8.77 × 10^16^
Glass/TiN (50 nm)	1.45 × 10^21^	7.22 × 10^15^
Glass/TiN (54.5 nm)	3.32 × 10^21^	1.81 × 10^16^
Glass/TiN (46 nm)/Inc. TiN (1.4 nm)	1.28 × 10^22^	5.87 × 10^16^
Glass/TiN (46 nm)/Inc. TiN (4.1 nm)	2.10 × 10^21^	9.64 × 10^15^
Glass/TiN (46 nm)/Inc. TiN (6.9 nm)	1.12 × 10^21^	5.13 × 10^15^
